# Low temperature, autotrophic microbial denitrification using thiosulfate or thiocyanate as electron donor

**DOI:** 10.1007/s10532-017-9796-7

**Published:** 2017-06-02

**Authors:** Elias Broman, Abbtesaim Jawad, Xiaofen Wu, Stephan Christel, Gaofeng Ni, Margarita Lopez-Fernandez, Jan-Eric Sundkvist, Mark Dopson

**Affiliations:** 1 Centre for Ecology and Evolution in Microbial Model Systems (EEMiS), Linnaeus University, Kalmar, Sweden; 2 Boliden Mineral AB, 936 81 Boliden, Sweden; 3 Department of Biology, University of Copenhagen, Universitetsparken 15, 2100 Copenhagen, Denmark

**Keywords:** Biodegradation, Eutrophication, Inorganic sulfur compound, Sulfide mineral, Wastewater

## Abstract

Wastewaters generated during mining and processing of metal sulfide ores are often acidic (pH < 3) and can contain significant concentrations of nitrate, nitrite, and ammonium from nitrogen based explosives. In addition, wastewaters from sulfide ore treatment plants and tailings ponds typically contain large amounts of inorganic sulfur compounds, such as thiosulfate and tetrathionate. Release of these wastewaters can lead to environmental acidification as well as an increase in nutrients (eutrophication) and compounds that are potentially toxic to humans and animals. Waters from cyanidation plants for gold extraction will often conjointly include toxic, sulfur containing thiocyanate. More stringent regulatory limits on the release of mining wastes containing compounds such as inorganic sulfur compounds, nitrate, and thiocyanate, along the need to increase production from sulfide mineral mining calls for low cost techniques to remove these pollutants under ambient temperatures (approximately 8 °C). In this study, we used both aerobic and anaerobic continuous cultures to successfully couple inorganic sulfur compound (i.e. thiosulfate and thiocyanate) oxidation for the removal of nitrogenous compounds under neutral to acidic pH at the low temperatures typical for boreal climates. Furthermore, the development of the respective microbial communities was identified over time by DNA sequencing, and found to contain a consortium including populations aligning within *Flavobacterium*, *Thiobacillus*, and *Comamonadaceae* lineages. This is the first study to remediate mining waste waters by coupling autotrophic thiocyanate oxidation to nitrate reduction at low temperatures and acidic pH by means of an identified microbial community.

**Electronic supplementary material:**

The online version of this article (doi:10.1007/s10532-017-9796-7) contains supplementary material, which is available to authorized users.

## Introduction

Wastewaters generated during mining and processing of metal sulfide ores can contain significant concentrations of nitrogenous compounds. These include nitrate (NO_3_^−^), nitrite (NO_2_^−^), and ammonium (NH_4_^+^) that are derived from nitrogen based explosives, such as ‘ammonium nitrate fuel oil’ (ANFO) and watergel-based explosives (Revey 1996). It has been reported that 0.2 to 28% of explosives are undetonated during blasting (best practice is 2–5% for ANFO) (Morin and Hutt 2009) and if the nitrogenous compounds are released, they cause eutrophication in water bodies. Some consequences of eutrophication are an increase in oxygen demand that results in recipient waters turning anoxic (Conley et al. 2011), possible hazards to human health (Knobeloch et al. 2000), and potential toxicity to aquatic animals (Alonso and Camargo 2003). In addition to nitrogenous pollutants, some mining wastewaters are rich in inorganic sulfur compounds (ISCs). These compounds can include toxic thiocyanate (SCN^−^) generated from cyanide used in gold extraction (Zagury et al. 2004). A further major ISC typically found in mining wastewaters is thiosulfate (S_2_O_3_^2−^) that can be generated from for example, hydrogen sulfide added during molybdenum flotation (Liljeqvist et al. 2011), ammoniacal thiosulfate leaching (Grosse et al. 2003), and dissolution of metal sulfides such as pyrite (Eq. 1) (Dopson and Johnson 2012; Schippers and Sand 1999). Complete oxidation of ISCs generates sulfuric acid (Eq. 2) that results in serious environmental damage if released due to acidification of recipient water bodies in a process termed ‘acid mine drainage’, abbreviated as AMD (Chen et al. 2016).

1 $${\text{FeS}}_{ 2} + {\text{ 6Fe}}^{ 3+ } + {\text{ 3H}}_{ 2} {\text{O}} \to {\text{S}}_{ 2} {\text{O}}_{ 3}^{ 2- } + {\text{ 7Fe}}^{ 2+ } + {\text{ 6H}}^{ + }$$

2 $${\text{S}}_{ 2} {\text{O}}_{ 3}^{ 2- } + {\text{ 8Fe}}^{ 3+ } + {\text{ 5H}}_{ 2} {\text{O}} \to {\text{ 2SO}}_{ 4}^{ 2- } + {\text{ 8Fe}}^{ 2+ } + { 1}0{\text{H}}^{ + }$$

A complicating factor in treating mining wastewaters in cold climates, such as in northern Sweden, is that the water temperature rarely increases above approximately 15 °C (Liljeqvist et al. 2011). Due to economic constraints, this requires the development of (bio)remediation processes for contaminant removal that operate at low temperatures.

High concentrations of nitrogen compounds such as NH_4_^+^ and NO_3_^−^ in mining waters can be treated by physical (e.g. membrane separation), chemical (e.g. electrochemical removal), and biological (e.g. nitrification and denitrification) methods (reviewed in Jermakka et al. 2015). Biological nitrogen removal typically consists of nitrification (NH_4_^+^ and NO_2_^−^ conversion to NO_3_^−^) followed by denitrification that couples oxidation of an electron donor [e.g. organic carbon, ferrous iron (Fe^2+^), or sulfide (S^2−^)] to NO_3_^−^ reduction that if completely reduced, eventually forms nitrogen gas (Lu et al. 2014). Recent work has confirmed that organic carbon compounds such as methanol and acetate favor specific denitrifying bacteria and successfully removes NO_3_^−^ (Li et al. 2016a). However, the use of methanol as an electron donor produces CaCO_3_, which increases the pH, and affects the denitrifying microbial community in the system. For example, it has been observed that up to 22.3% of the NO_3_^−^ concentration in a methanol fed bioreactor accumulated as NO_2_^−^ while increasing the pH up to 9.2. In contrast, at pH 7.6 the accumulation of NO_2_^−^ was negligible (Li et al. 2016b). Biological nitrogen removal in wetlands or reactive barriers functions by promoting denitrification processes in sediments or buried organic matter, respectively (Batty and Younger 2004). Nitrogen removal has also been demonstrated in bioreactors with organic (e.g. Papirio et al. 2014; Zou et al. 2014) and inorganic electron donors including ISCs (e.g. Chung et al. 2014; Di Capua et al. 2015; Zhang et al. 2009; Zou et al. 2016). Other studies investigating S_2_O_3_^2−^ oxidation coupled with NO_3_^−^ reduction utilized pure cultures of *Thiobacillus denitrificans* (Justin and Kelly 1978a, b) and *Sulfurimonas denitrificans* [originally named *Thiomicrospira denitrificans* (Hoor 1975)]. SCN^−^ oxidation can also be linked to NO_3_^−^ reduction by the soda-lake sediment dwelling species *Thialkalivibrio thiocyanodenitrificans* (Sorokin et al. 2004). In some microorganisms, SCN^−^ can act as an electron donor as well as providing sulfur and nitrogen to the cell (Ogawa et al. 2013). Finally, a novel SCN^−^ degrading pathway has been identified by metagenomics in a *Thiobacillus*-like strain (Kantor et al. 2015). Advantages of ISC fed autotrophic versus heterotrophic denitrification are that less sludge is produced and costs are reduced as an organic electron donor does not need to be added. A disadvantage is the reduced pH due to generation of sulfuric acid (Eq. 2). However, efficient autotrophic, ISC-fed, low temperature denitrification systems for thiocyanate removal remain to be developed.

To be able to increase production from sulfide mineral mining coupled to more stringent regulatory limits on the release of ISCs and NO_3_^−^, low cost techniques to remove these pollutants under ambient temperatures are required. In this study, autotrophic ISC oxidation coupled to removal of nitrogenous compounds was investigated at low temperature. In addition, the structures of the microbial communities were identified by DNA sequencing.

## Materials and methods

### Bioreactor set-up

Continuous culture bioreactors (500 mL working volume) were operated with a basal salts medium plus nitrate as electron acceptor. The feed medium was autoclaved before filter sterilized (0.2 µm membrane filter; Sarstedt, Nümbrecht, Germany) ISCs were added, i.e. thiosulfate (0.89 mM) for a thiosulfate reactor and thiocyanate (6.88 mM) for the aerobic and anaerobic reactors (Table 1). For both experiments (described below), the retention time was 9 h and the initial pH in the bioreactors was adjusted to 8.0–8.5 by addition of 1 M Na_2_CO_3_. Microbial biomass was retained in the reactors by promoting biofilm growth on 50% (vol/vol) autoclaved high surface area biofilm carriers (AnoxKaldnes). The bioreactors and medium feed were maintained at the desired temperature by placing them in a constant temperature room.

**Table 1:** Contents of the medium used for the two types of bioreactors

Compound	Nutrient	S_2_O_3_ R3 bioreactor (mg L^−1^)	SCN^−^ R1 & R2 bioreactors (mg L^−1^)
Mg(SO_4_)		25	25
KCl		5	5
K_2_HPO^4^		25	25
(NH4)_2_SO_4_	NH_4_^+^	8.17	8.17
Ca(NO_3_)_2_*	NO_3_^−^	113.3	2010
Na_2_S_2_O_3_	S_2_O_3_^2−^	100 (0.89 mM)	
KSCN	SCN^−^		399.97 (6.88 mM)

If a nutrient is specified with the compound name the concentration indicates the amount of nutrient in the medium

* or Ca(NO_3_)_2_·4H_2_O

### Thiocyanate fed bioreactors

Two SCN^−^ fed bioreactors were operated between June 3rd 2014 and November 9th 2015 (totaling 524 days; Table 2). One of the bioreactors was maintained aerobic (referred to as R1) and the other anaerobic (R2) by sparging with 300 mL sterile filtered air and N_2_-gas (0.2 µm Acro 50 air filter; PALL Corporation), respectively. The reactors were inoculated with water sampled from a municipal wastewater treatment plant (Tuvanverket), Skellefteå, Sweden. The temperature in the bioreactors was lowered over time: day 0–231 at 21 °C; 232–300 at 15 °C; 301–337 at 10 °C; followed by 8 °C until the end of the experiment. In addition, the pH was lowered with 4.6 M H_2_SO_4_ to 5.5 at day 448 and then to 3.8 at day 510.

**Table 2:** An overview of the experimental design of the aerobic R1 and anaerobic R2 SCN^−^ fed bioreactors

Days	°C	pH
0–231	21	8.0–8.5
232–300	15	8.0–8.5
301–338	10	8.0–8.5
339–448	8	8.0–8.5
449–510	8	5.5
511–524	8	3.5

Temperature and pH were lowered throughout the experiment

### Thiosulfate fed bioreactor

The S_2_O_3_^2−^ fed bioreactor (R3) was maintained at 8 °C and operated between February 11th 2013 and December 16th 2013 (308 days) in microaerophilic conditions i.e. the reactor was not sparged with air and the medium was stirred gently with a magnetic flea such that the surface of the medium was not broken. The bioreactor was inoculated with a sediment sample removed from an AMD stream 250 m below ground in the Boliden operated Kristineberg mine, Sweden. Details of the AMD stream including the microorganisms present (but not from the underlying sediment used here) are available (Liljeqvist et al. 2015).

### Chemical measurements

Measurements for chemical parameters were conducted by opening and sampling the bioreactors. The R1 and R2 SCN^−^ fed bioreactors were measured for pH, redox potential, chemical oxygen demand (COD), NO_2_^−^, NO_3_^−^, NH_4_^+^ and SCN^−^ while the R3 bioreactor fed with S_2_O_3_^2−^ was measured for pH, redox potential, NO_3_^−^, protein concentration, S_2_O_3_^2−^, and tetrathionate (S_4_O_6_^2−^). pH and redox potential were measured using electrodes (pHenomenal, VWR pH electrode and Ag/AgCl SI Analytics electrode, Mettler Toledo, respectively). Protein concentration was measured using the Bio-Rad protein assay (Bradford protein assay, Bio-Rad). COD (LCI 500), NO_2_^−^ (LCK 342), NO_3_^−^ (LCK 339), and NH_4_^+^ (LCK303) were measured using Hach Lange kits according to the manufacturer’s instructions. S_4_O_6_^2−^ and S_2_O_3_^2−^ were measured by forming an iron-SCN^−^complex according to Kelly et al. (1969) with the modification of using an ammonium/acetate buffer before the addition of the iron nitrate (Dopson and Lindström 1999). SCN^−^ was measured as described for S_4_O_6_^2−^ and S_2_O_3_^2−^ except that the step of adding cyanide was omitted.

### DNA extraction, RFLP, and DNA sequencing of the S_2_O_3_^2−^ bioreactor inoculum

The inoculum of the S_2_O_3_^2−^ fed R3 bioreactor was sampled for DNA extraction by centrifuging 10 mL bioreactor content at 10,000×*g* for 10 min. The resulting pellet was washed with 100 mM Tris HCl and 10 mM EDTA and re-suspended in 10 mM Tris HCl and 1 mM EDTA. Cells were lysed and genomic DNA extracted according to Dopson and Lindström (2004) and Morales et al. (2005) before the PCR fragments were analyzed by restriction fragment length polymorphism (RFLP) as described in Wu et al. (2013). Briefly, a portion of the bacterial 16S rRNA gene was amplified using the primers GM5F and 907R (Muyzer et al. 1995), the generated PCR fragments were cleaned using a QIAquick PCR Purification Kit (Qiagen), cloned using the pGEM-T Easy Vector System (Promega), and transformed (Hanahan 1983) into *Escherichia coli* strain DH5α (Sambrook et al. 1989). The resulting transformants were plated onto Luria–Bertani (LB) plates and plasmid DNA extracted using the QIAGEN Plasmid Mini Kit. The plasmids were individually cut using the restriction enzymes HhaI and MspI and fragments separated by agarose gel electrophoresis. Restriction fragment patterns were identified and representative unique clones were Sanger sequenced in both directions by Macrogen. Obtained sequences were checked using DECIPHER (Wright et al. 2012), edited in Geneious version 6.0.6 (Biomatters Ltd Auckland, New Zealand), and compared with the NCBI GenBank database. A phylogenetic tree was created with Molecular Evolutionary Genetic Analysis version 5.1 (Tamura et al. 2011). 16S rRNA gene sequences were submitted to GenBank with the BioProject accession number PRJNA347259.

### DNA extraction, high throughput 16S rRNA gene tag sequencing, and bioinformatics

At the end of the experiment (day 308), a sample of the microbial community attached to biofilm carriers in the S_2_O_3_^2−^ fed R1 and R2 bioreactors was detatched by vortexing several biofilm carrier pellets in 5 mL culture medium. DNA was extracted and high-throughput sequencing (Illumina MiSeq) performed as described below. In addition, cells (50 mL culture) from the aerobic R1 and anaerobic R2 SCN^−^ fed reactor inocula were filtered onto a 25 mm 0.2 µm pore-size filter (Supor-200, PALL Corporation). Finally, the microbial communities in the two SCN^−^ fed reactors were sampled (one biofilm carrier for each reactor at each ocassion) for DNA extraction and second generation sequencing as described for the S_2_O_3_^2−^ fed bioreactor biofilm carrier pellets.

DNA to be sequenced using high-throughput Illumina methodology was extracted using the PowerWater DNA Isolation Kit (MO BIO Laboratories) and partial 16S rRNA genes amplified with a modified PCR protocol by Hugerth et al. (2014) using primers 341F and 805R (Herlemann et al. 2011). The PCR amplification and Illumina libraries were constructed and sequenced at Science for Life Laboratory, Sweden (www.scilifelab.se) according to Lindh et al. (2015). The UPARSE pipeline (Edgar 2013) was used to process the sequencing data. Clustered OTUs were annotated against the SINA/SILVA database (SILVA 119; Quast et al. 2013), and finally analyzed in Explicet 2.10.5 (Robertson et al. 2013). The amount of pair-end reads received from the sequencing facility, merged and quality trimmed reads, and amount of OTUs clustered can be found in Supplemental File 1. Sequences for phylogenetic analyses were aligned with ClustalW, and built in MEGA 7 as maximum likelihood trees (Kumar et al. 2016). 16S rRNA gene sequences were submitted to GenBank with the BioProject accession number PRJNA347259.

## Results

### Aerobic SCN^−^ fed bioreactor

**Fig. 1:**
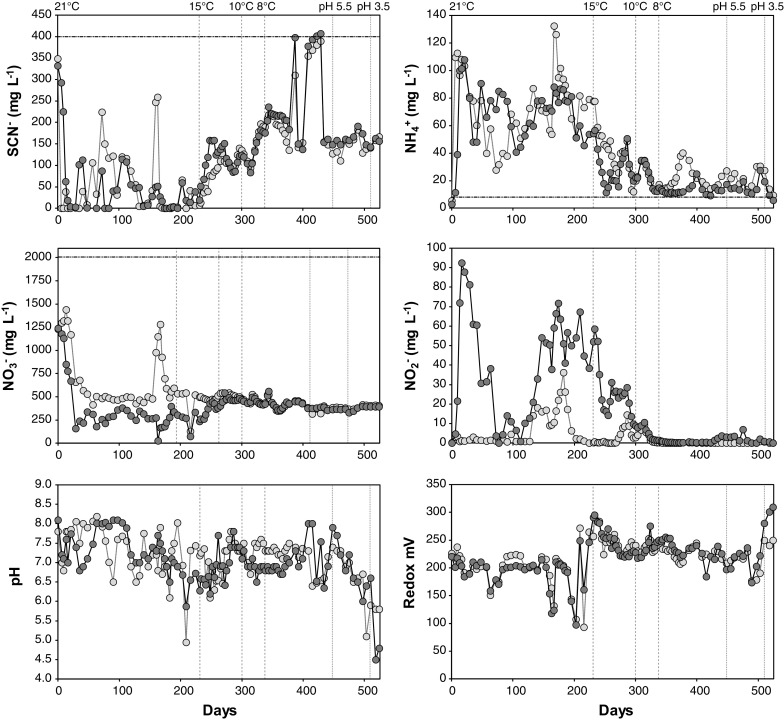
Chemical measurements from the aerobic R1 (*light grey circles*) and anaerobic R2 (*dark grey circles*) SCN^−^ fed bioreactor adapted from 21 °C room temperature to 8 °C over a period of 338 days. The pH was lowered from 8.0–8.5 to 5.5 on day 448 and further lowered to pH 3.8 on day 510. The *horizontal black line* denotes the input concentration in the medium (electron donor SCN^−^, electron acceptor NO_3_^−^, and NH_4_^+^). One SCN^−^ outlier value (751.5 mg L^−1^) has been removed for the anaerobic bioreactor on day 57

**Fig. 2:**
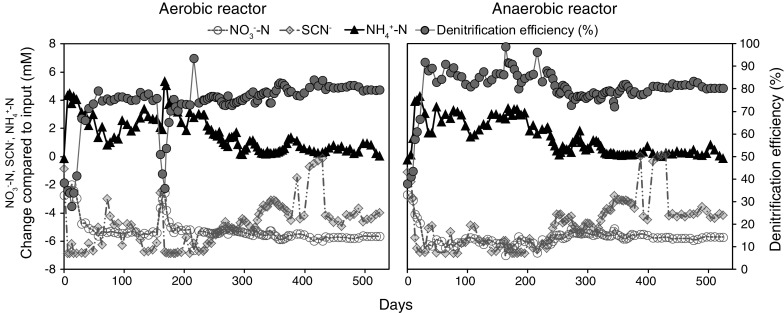
Changes in NO_3_^−^–N, SCN^−^, and NH_4_^+^–N concentrations over time compared to the input (7.1, 6.8, and 0.4 mM, respectively) in the aerobic R1 and anaerobic R2 SCN^−^ fed bioreactor. Denitrification efficiency (% NO_3_^−^–N removed from input) is shown on the right *y*-axis. One outlier SCN^−^ value (+6.1 mM) was removed for the anaerobic bioreactor on day 57

Chemical measurements were conducted by sampling the bioreactor. The aerobic SCN^−^ fed R1 bioreactor had SCN^−^ concentrations of 50.8 ± 72.6 mg L^−1^ (number of data points (*n*) = 37; ± 1 SD). This corresponded to a 85.2 ± 21.5% decrease in electron donor, i.e. 5.8 ± 1.5 mM. This was the case during most measuring points until the temperature was decreased below 15 °C when the SCN^−^ concentration increased gradually and remained at 152.6 ± 86.9 mg L^−1^ (*n* = 51; 62.5 ± 12.9% decrease of electron donor, i.e. 4.3 ± 0.9 mM) until pH was decreased. The NO_3_^−^ concentration in the aerobic bioreactor was initially 1230 mg L^−1^ and after 21 days it had decreased and stabilized at 548.8 ± 176.4 mg L^−1^ (*n* = 33; 71.0 ± 10.9% decrease of electron acceptor, i.e. 22.9 ± 3.5 mM) until the temperature was lowered to 15 °C. After the temperature decrease, the NO_3_^−^ concentration remained at 436.2 ± 64.0 mg L^−1^ (*n* = 51; 77.2 ± 2.0% decrease of electron acceptor, i.e. 24.9 ± 0.6 mM) until pH decrease, with a following slight decrease after pH was lowered. Most of the time in the aerobic reactor the NO_2_^−^ concentration was below 10 mg L^−1^, except peaking between day 133-189 at 18.4 ± 14.2 mg L^−1^ (*n* = 13; Fig. 1). The concentration of NH_4_^+^ was 75.2 ± 25.4 mg L^−1^ (*n* = 38) until it gradually dropped within a range of 14–49 mg L^−1^ after the temperature was lowered to 15 and 10 °C on day 231 and 300, respectively (Fig. 1). Pearson correlations from the aerobic bioreactor showed that NH_4_^+^ positively correlated with NO_3_^−^ and NO_2_^−^ (p < 0.01; r = 0.592 and 0.519, respectively). For a full list of Pearson correlations see Supplemental File 2. The calculated denitrification (NO_3_^−^–N removal) efficiency in the aerobic reactor was typically in the range of 70–80% throughout the experiment (Fig. 2). Initially, the bioreactor had a pH of 7.8 and remained at 7.2 ± 0.5 until the pH was decreased with H_2_SO_4_ to 5.5 and 3.5 at day 448 and 510, respectively (*n* = 89; Fig. 1). The redox potential remained at 198.5 ± 35.9 mV until the temperature was decreased to 15 °C on day 231 (*n* = 39). At this point, the redox increased and remained at 233.6 ± 20.7 mV until the end of the experiment (*n* = 63; Fig. 1). Optical density (460 nm) in the water phase remained low throughout the experiment (<0.01 abs) while COD had a high variation with values of 3–462 mg L^−1^ before the temperature was decreased to 15 °C and then it eventually stabilized after the pH was decreased to 5.5 at 172–248 mg L^−1^ (Supplemental File 3).

**Fig. 3:**
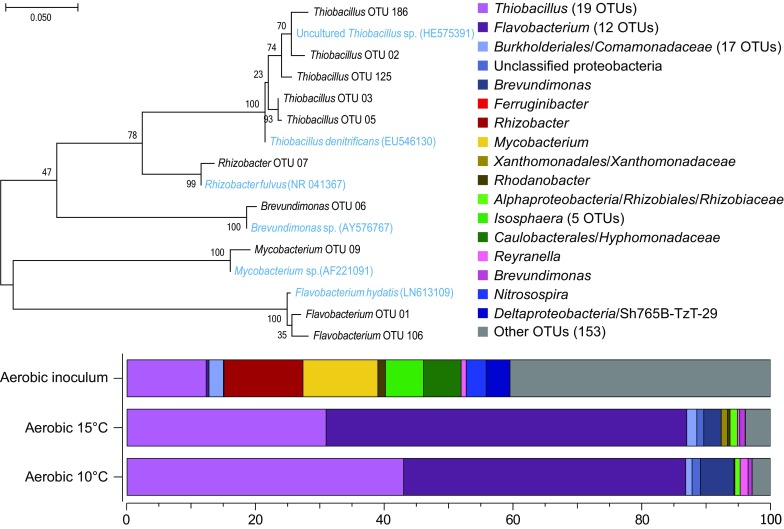
Phylogenetic tree of the dominant OTUs in the aerobic SCN^−^ fed R1 bioreactor and stacked bars of single OTUs. The tree with the highest maximum log likelihood (−2032.68) is shown, alongside close relative species (*blue* text) obtained from NCBI GenBank. DNA was extracted and the 16S rRNA gene was sequenced; from the inoculum; at day 300 after 69 days at 15 °C; and at day 338 after 38 days at 10 °C. The grouping “Other OTUs” comprises OTUs with less than 1% total relative abundance (among all samples summed)

The molecular phylogeny of the R1 bioreactor inoculum as well as at the end of the 15 and 10 °C temperature changes was investigated (Fig. 3). A full list of annotated OTUs with relative abundance can be found in Supplemental File 4. The aerobic inoculum was dominated by the genera *Rhizobacter* (relative abundance of 12.7%), *Thiobacillus* (12.3%), *Mycobacterium* (11.5%), *Isosphaera* (5.9%), and the family *Hyphomonadaceae* (5.8%). After the temperature had been lowered to 15 °C for 69 days the microbial community in the aerobic bioreactor had changed to consist mainly of the genera *Flavobacterium* (56.0%) and *Thiobacillus* (30.9%). This high relative abundance of the genus *Thiobacillus* was further increased after the temperature had been lowered to 10 °C for 38 days. The aerobic microbial community was then dominated by the genera *Flavobacterium* (43.8%) and *Thiobacillus* (43.0%). Phylogenetic analysis of closely related species downloaded from NCBI GenBank indicated that the *Thiobacillus* OTUs from the 16S rRNA gene data were most closely related to *T. denitrificans*; the *Rhizobacter* OTU was indicated to be related to *Rhizobacter fulvus*; and the *Flavobacterium* OTUs to *Flavobacterium hydatis* (Fig. 3).

### Anaerobic SCN^−^ fed bioreactor

The SCN^−^ concentration in the anaerobic R2 bioreactor was 44.9 ± 63.5 mg L^−1^ (*n* = 36; corresponding to a 82.1 ± 34.1% decrease of electron donor, i.e. 5.6 ± 2.3 mM) until the temperature was decreased below 15 °C when the SCN^−^ concentration gradually increased and remained at 170.2 ± 88.9 mg L^−1^ (*n* = 51; 59.1 ± 13.7% decrease of electron donor, i.e. 4.0 ± 0.9 mM) until pH was decreased. This pattern starting with the temperature decrease to 15 °C was also observed for COD and NO_3_^−^, and a decrease in NH_4_^+^ and NO_2_^−^ (Fig. 1). These changes were confirmed with Pearson correlations for the chemical parameters in the anaerobic vessel which showed that SCN- positively correlated with COD and NO_3_^−^ (p < 0.01; r = 0.468 and 0.329, respectively). While negatively correlating with NH_4_^+^ and NO_2_^−^ (p < 0.01; r = − 0.617 and −0.530, respectively).

The NO_3_^−^ concentration in the anaerobic R2 bioreactor showed a similar pattern to that of the aerobic R1 reactor, but concentrations remained lower. The initial NO_3_^−^ concentration at 21 °C was 1240 mg L^−1^ that decreased over the initial 21 days and stabilized at 317.7 ± 110.3 mg L^−1^ (*n* = 53; 86.9 ± 4.2% decrease of electron acceptor, i.e. 28.0 ± 1.3 mM) until the temperature was lowered below 15 °C. After the temperature decrease, the NO_3_^−^ concentration remained at 419.5 ± 64.3 mg L^−1^ (*n* = 51; 78.8 ± 2.7% decrease of electron acceptor, i.e. 25.4 ± 0.9 mM) until pH was lowered. This lower NO_3_^−^ concentration in the anaerobic R2 reactor at 21 °C indicated a more efficient reduction of NO_3_^−^ compared to the aerobic R1 reactor. This difference between the samples could not be seen after the temperature and pH had been lowered (i.e. below 15 °C and pH 8). Up until day 300, the NO_2_^−^ concentrations in the anaerobic R2 reactor showed the opposite pattern compared to the aerobic R1 reactor and varied from low (~1 mg L^−1^) to high (92.4 mg L^−1^) but with an overall high concentration of 35.6 ± 23.6 mg L^−1^ (*n* = 59). This difference between the R1 and R2 reactors disappeared when the temperature was lowered to 10 °C on day 300 (Fig. 1). The NH_4_^+^ concentration in the anaerobic R2 reactor showed a similar pattern to the aerobic R1 bioreactor. Concentrations remained at 64.7 ± 23.4 mg L^−1^ (*n* = 38) until it gradually dropped to a range of 9–51 mg L^−1^ after the temperature was lowered to 15 and 10 °C (*n* = 30; Fig. 1). Pearson correlations from the anaerobic R2 reactor showed that NH_4_^+^ correlated positively with NO_2_^−^ (p < 0.01; r = 0.774) while negatively correlating with NO_3_^−^ (p < 0.01; r = −0.280). The calculated denitrification efficiency in the anaerobic R2 reactor was typically in the range of 70–90% throughout the experiment (Fig. 2). pH measurements were similar to the aerobic R1 reactor throughout the experiment, with an initial pH of 8.1 that remained in the range of 7.1 ± 0.5 until the pH was decreased with H_2_SO_4_ (*n* = 89; Fig. 1). The redox potential showed a similar pattern to the aerobic R1 reactor and remained at 191.9 ± 31.4 mV until the temperature was decreased to 15 °C on day 231 (*n* = 39). The redox then remained at 235.7 ± 23.3 mV until the pH was decreased to 3.5 at day 510 causing the redox to gradually increase to 309.3 mV (*n* = 60; Fig. 1). Optical density (460 nm) in the water phase remained low throughout the experiment (<0.01 abs) and COD in the anaerobic R2 bioreactor showed a similar pattern to the aerobic R1 reactor, with a trend to gradually increase during the experiment and stabilize the longer the experiment progressed. COD was measured with a high variation throughout the experiment (8–466 mg L^−1^) before the 15 °C temperature decrease, to eventually stabilize after pH was decreased to 5.5 at 198–250 mg L^−1^ (Supplemental File 3).

**Fig. 4:**
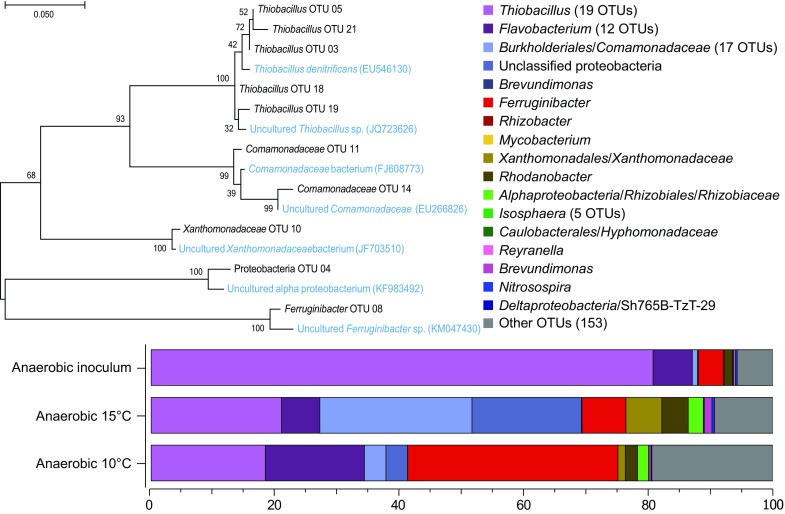
Phylogenetic tree of the dominant OTUs in the anaerobic SCN^−^ fed R2 bioreactor and stacked bars of single OTUs. The tree with the highest maximum log likelihood (−1977.7296) is shown, alongside close relative species (*blue* text) obtained from NCBI GenBank DNA was extracted and the 16S rRNA gene was sequenced; from the inoculum; at day 300 after 69 days at 15 °C; and at day 338 after 38 days at 10 °C. The grouping “Other OTUs” comprises OTUs with less than 1% total relative abundance (among all samples summed)

The molecular phylogeny of the anaerobic R2 bioreactor inoculum as well as at the end of the 15 and 10 °C temperature changes was investigated (Fig. 4) and a full list of annotated OTUs plus relative abundance is provided in Supplemental File 4. The anaerobic inoculum mainly consisted of the genera *Thiobacillus* with a relative abundance of 80.7%, *Flavobacterium* (6.3%), and *Ferruginibacter* (4.6%). After 69 days at 15 °C, the community was dominated by the family *Comamonadaceae* (24.5%) plus the genera *Thiobacillus* (20.9%), *Ferruginibacter* (7.1%), *Flavobacterium* (6.2%), and one OTU annotated as an unclassified proteobacteria (17.6%). After the decrease to 10 °C, the community mainly consisted of the genera *Ferruginibacter* (34.8%), *Thiobacillus* (18.4%), and *Flavobacterium* (15.9%). Phylogenetic analysis of close relative species downloaded from NCBI GenBank indicated that the *Thiobacillus* OTUs from the 16S rRNA gene data were most closely related to *T. denitrificans* (Fig. 4).

### Microaerophilic S_2_O_3_^2−^ fed bioreactor

**Fig. 5:**
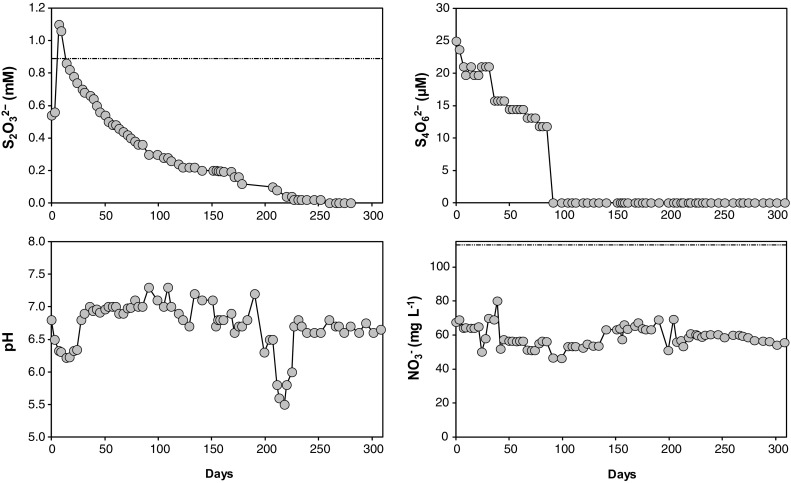
Chemistry measurements from the microaerophilic S_2_O_3_^2−^ fed R3 bioreactor at 8 °C. The *horizontal black line* denotes the input concentration in the medium (electron donor: S_2_O_3_^2−^ and electron acceptor NO_3_^−^)

In the S_2_O_3_^2−^ fed R3 bioreactor, the S_2_O_3_^2−^ concentration in the vessel gradually decreased during the experiment from 1.1 mM on day 7 to 0 mM after 266 days. The S_4_O_6_^2−^ concentration in the vessel gradually decreased throughout the experiment from 19.7 to 0 µM on day 91 suggesting little S_4_O_6_^2−^ accumulated from S_2_O_3_^2−^ oxidation. However, a concomitant decrease in NO_3_^−^ was not observed in relation to the decrease of S_2_O_3_^2−^ and the concentration of NO_3_^−^ concentration was stable at 59.0 ± 6.2 mg L^−1^ throughout the experiment (*n* = 69; Fig. 5; corresponding to a 48.6 ± 8.2% decrease of electron acceptor, i.e. 0.9 ± 0.2 mM). However, this concentration was less than the 113.3 mg L^−1^ in the influent potentially due to denitrification. The pH remained at 6.7 ± 0.4 throughout the experiment (n = 69; Fig. 5) while the redox potential gradually decreased from 203.3 to 50.6 mV for the first 154 days when it sharply increased to 220.5 mV. This increase was probably due to the stirring in the vessel being too high and hence, introducing more oxygen into the vessel. The protein concentration was stable throughout the whole experiment and remained at 10.6 ± 0.44 µg mL^−1^ (*n* = 68) expect during the first day (31.2 µg mL^−1^) when the experiment started (Supplemental File 5).

**Fig. 6:**
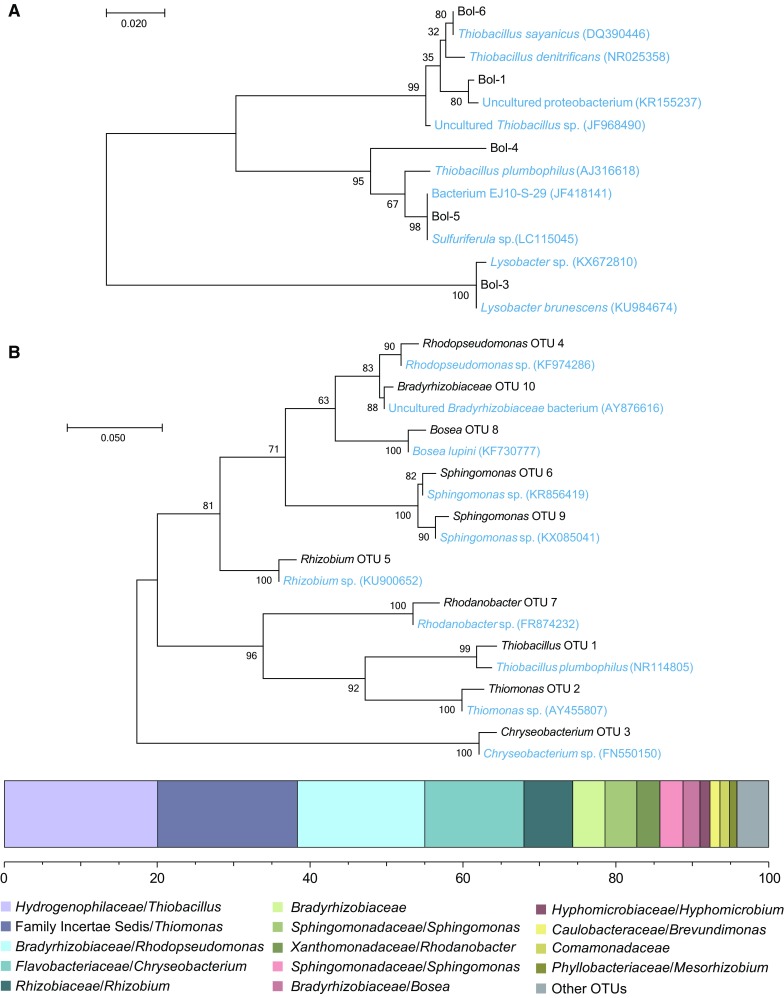
Identified partial 16S rRNA genes in the microaerophilic S_2_O_3_^2−^ fed R3 bioreactor inoculum culture and at the end of the experiment. **a** Phylogenetic tree of species compared to the inoculum from RFLP analysis, samples derived from unique restriction fragment patterns are labelled as Bol-1 to 6. **b** Phylogenetic tree and stacked bar chart of the dominant OTUs in the microaerophilic S_2_O_3_^2−^ fed bioreactor at the end of the experiment after 308 days. The tree with the maximum highest log likelihood (−2332.68) is shown, alongside close relative species (*blue* text) obtained from NCBI GenBank. In the stacked bar chart single OTUs are shown as family/genus (two technical replicates). The grouping “Other OTUs” comprises OTUs with less than 1% total relative abundance (among all samples summed)

The inoculum of the S_2_O_3_^2−^ fed R3 reactor was sequenced after RFLP analysis and sequences derived from unique bands from the electrophoresis gel were aligned with known sequences (Fig. 6a). The results indicated that the inoculum consisted of *T. denitrificans*-like species and/or iron-reducing bacteria. Other sequences clustered tightly with unclassified *Gammaproteobacteria* and *Lysobacter brunescens*. DNA extracted from the end of the experiment after 308 days was sequenced on the Illumina platform and showed a microbial community consisting of highly abundant single OTUs belonging to the genera *Thiobacillus* with a relative abundance of 20.1 ± 0.7% (*n* = 2, two technical replicates), *Thiomonas* (18.3 ± 0.1%), *Rhodopseudomonas* (16.7 ± 1.3%), *Cryseobacterium* (13.0 ± 0.4%), *Rhizobium* (6.4 ± 0.3%), and other lower abundant OTUs (Fig. 6b). A full list of high throughput sequenced OTUs with relative abundance can be found in Supplemental File 6. Phylogenetic analysis of close relative species downloaded from NCBI GenBank indicated that the *Thiobacillus* OTU was most closely related to *Thiobacillus plumbophilus* and the *Bosea* OTU to *Bosea lupini* (Fig. 6b).

## Discussion

The aerobic SCN^−^ fed R1 bioreactor simulated a pre-denitrification chamber with the goal of oxidizing the SCN^−^ via ammonia/ammonium formation to NO_2_^−^ and NO_3_^−^ (Eqs. 1–7 in Supplemental File 7) before an anaerobic denitrification chamber. Results from the aerobic R1 bioreactor showed that SCN^−^ and NO_3_^−^ were removed throughout the experiment, but the concentration of NO_2_^−^ and NO_3_^−^ was higher at room temperature, potentially due to ongoing nitrification. In conjunction with this decrease of SCN^−^ and NO_3_^−^, an increase was observed for NH_4_^+^ when operated at room temperature (Figs. 1, 2). Three suggested reaction schemes for this process are: (i) formation of NH_4_^+^ by direct aerobic oxidation of SCN^−^ (Eq. 1 in Supplemental File 7); (ii) aerobic oxidation of SCN^−^ to cyanate (CNO^−^) followed by NH_4_^+^ formation by CNO^−^ hydrolysis (Eqs. 2–3 in Supplemental File 7) which increases rapidly with a decrease in pH (Warner 1942); or (iii) microbial dissimilatory reduction of NO_3_^−^ into NH_4_^+^ and NO_2_^−^ or N_2_ inside the biofilm (Eqs. 4 and 5 in Supplemental File 7). The data showed an approximate 1:4 fold molar ratio of SCN^−^:NO_3_^−^ reduction and therefore, suggesting microbial dissimilatory reduction occurred (Eq. 4 in Supplemental File 7). Removal of SCN^−^ in conjunction with NO_3_^−^ reduction has been observed previously with the use of biomaterial from a biogas plant (Sahariah and Chakraborty 2012), while dissimilatory nitrate reduction into ammonia has previously been observed in an industrial system operated at 22–28 °C (Villemur et al. 2015). The diverse microbial community attached to the biofilm carriers became dominated by *Flavobacterium*-like and *Thiobacillus*-like OTUs (related to *T. denitrificans*; Fig. 3). *Flavobacterium spp.* include facultative anaerobic species able to reduce NO_3_^−^, e.g. *Flavobacterium denitrificans* (Horn et al. 2005). The genus *Thiobacillus* contains species with a versatile lifestyle that are able grow under aerobic and anaerobic conditions with a wide variety of electron donors such as SCN^−^ (Villemur et al. 2015), S_2_O_3_^2−^ (Trouve and Chazal 1999), and H_2_S (Subletta 1987) along with NO_2_^−^ or NO_3_^−^ as an electron acceptor (Justin and Kelly 1978a). Considering that oxygen decreases the growth rate of e.g. *T. denitrificans* (Subletta 1987), we suggest that part of the microbial community carried out nitrate removal under microaerophilic/anaerobic conditions that are well-known to develop inside biofilms formed on carrier material (Munn 2011). However, compared to laboratory studies conducted at 30 °C, here we have shown that removal of SCN^−^ and NO_3_^−^ also occurred in oxic wastewater with the use of biofilm carriers at 20, 15, and 10 °C which are typical for boreal climates. We also observed removal of SCN^−^ and NO_3_^−^ after the pH was lowered to pH 5.5 and 3.5. These data indicated that the microbial community carried out these processes during acidic conditions which are typical for some mining wastewaters.

The anaerobic SCN^−^ fed R2 bioreactor simulated a denitrification chamber following the aerobic pre-denitrification treatment. Such chambers are common in wastewater treatment plant designs typically consisting of an anaerobic denitrification chamber followed by a cycling of the wastewater in anoxic and oxic chambers (Lu et al. 2014). Similar to the aerobic R1 bioreactor, ${\text{NO}}_{ 3}^{ - }$ and SCN^−^ were removed from the systems but compared to the aerobic R1 reactor no increase of NO_3_^−^ was seen at room temperature (Fig. 1). A possible reaction for this is NO_3_^−^ reduction to N_2_-gas in conjunction with SCN^−^ oxidation to CNO^−^ (Eqs. 8 and 9 in Supplemental File 7), followed by hydrolysis of CNO^−^ and NH_4_^+^ formation (Eq. 3 in Supplemental File 7). Also similar to the aerobic R1 reactor, SCN^−^ and NO_3_^−^ were also removed after the pH had been lowered to 3.5. This was likely as nitrification was inhibited. Compared to the aerobic R1 bioreactor, the NO_2_^−^ concentration was higher at room temperature, indicating a more efficient microbial denitrification in the anaerobic reactor. Similar to the aerobic R1 bioreactor, NH_4_^+^ concentration also increased in conjunction with a decrease of SCN^−^ and NO_3_^−^ (Fig. 2). Through the experiment the microbial community on the biofilm carriers changed from being dominated by *Thiobacillus*-like OTUs to a more diverse community consisting of *Comamonadaceae*-like, *Thiobacillus*-like*, Flavobacterium*-like, and *Ferruginibacter*-like OTUs (Fig. 4). In addition to species within the *Thiobacillus* genus, *Comamonadaceae* are able to reduce nitrate (Khan et al. 2002) and *Flavobacterium* spp. grow on NO_2_^−^ (Pichinoty et al. 1976). Compared to the aerobic SCN^−^ fed R1 bioreactor the anaerobic R2 reactor had a higher variety of dominant microbial genera, potentially due to higher nitrate removal providing increased concentrations of intermediate nitrogen compounds. All known species belonging to the genus *Ferruginibacter* are strictly aerobic and incapable of nitrate reduction (Kang et al. 2015; Lee et al. 2014; Lim et al. 2009). Consequently, in this study a potentially novel *Ferruginibacter*-like population (Fig. 4) was selected that has an unknown role in the anaerobic SCN^−^ fed R2 bioreactor. To our knowledge this is the first study using next-generation sequencing to analyze the microbial community composition in SCN^−^ fed reactors operated at cold temperatures.

In the S_2_O_3_^2−^ fed R3 bioreactor the concentrations of S_2_O_3_^2−^ and NO_3_^−^ were always below input levels (Fig. 5) and indicate thiosulfate oxidation in conjunction with nitrate reduction. Similar findings have previously been reported and this process has found to be conducted by species *T. denitrificans* (Chung et al. 2014; Oh et al. 2000; Trouve and Chazal 1999; Zhang et al. 2009; Zou et al. 2016). Along with *Thiobacillus* spp., species from the *Thiomonas* genus are known to oxidize S_2_O_3_^2−^ (Chen et al. 2004) and these populations became abundant in the S_2_O_3_^2−^ fed R3 bioreactor (Fig. 6). It can therefore be concluded that our experimental set-up supported S_2_O_3_^2−^ oxidation coupled to NO_3_^−^ reduction and was comparable to previous studies. In addition, the second generation sequencing (Illumina MiSeq) at the end of the experiment showed a higher diversity of taxa compared to that of the RFLP analysis used to sequence the inoculum. This is in accordance to previous studies using second generation sequencing which has a greater power to reveal the complete diversity of microbial taxa (Sinclair et al. 2015).

Removal of NO_3_^−^ and ISC in mining wastewaters is feasible (Di Capua et al. 2015) and industrial systems are under development, such as the SCN^−^ removal in the LaRonde gold mine, Canada (Villemur et al. 2015). In this study, we have shown a potential to denitrify mining wastewater with the use of SCN^−^ as an electron donor, and that this can be achieved at sub-optimal temperatures such as 10–15 °C under both oxic and anoxic conditions from pH 8 down to 3.5. Many of these reactions were potentially carried out under microaerophilic/anaerobic conditions inside the biofilm on the biofilm carriers. However, our experiment produced large amounts of NH_4_^+^ from this process which would need to undergo further nitrification. It is suggested that following SCN^−^ depletion a re-cycling of the wastewater through oxic and anoxic conditions should be conducted. This would hypothetically nitrify the produced NH_4_^+^ followed by anaerobic denitrification of produced NO_3_^−^. The room temperature SCN^−^ and NO_3_^−^ removal rates were comparable to a published study carried out at mesophilic temperatures (Sahariah and Chakraborty 2012) but additionally, we report efficient SCN^−^ and NO_3_^−^ removal at temperatures typical for the boreal climate (15 and 10 °C) and a pH typical for mining wastewaters (Table 3).

**Table 3:** An overview of denitrification efficiency (%) from this study in the aerobic R1 and anaerobic R2 SCN^−^ fed bioreactors at different temperatures and pH values

Study	Condition	°C	pH	SCN^−^ influent (mg/L)	NO_3_^−^ influent (mg/L)	SCN^−^/NO_3_^−^ ratio	Denitrification efficiency (%)	Measuring points (*n*)
This study	Aerobic	21	8.0–8.5	400	2010	0.2	65.5 ± 16.6	39
This study	Aerobic	15	8.0–8.5	400	2010	0.2	75.0 ± 1.4	19
This study	Aerobic	10	8.0–8.5	400	2010	0.2	76.8 ± 1.7	11
This study	Aerobic	8	8.0–8.5	400	2010	0.2	79.8 ± 2.8	22
This study	Anaerobic	21	8.0–8.5	400	2010	0.2	81.4 ± 14.2	39
This study	Anaerobic	15	8.0–8.5	400	2010	0.2	79.7 ± 4.3	19
This study	Anaerobic	10	8.0–8.5	400	2010	0.2	77.4 ± 1.3	11
This study	Anaerobic	8	8.0–8.5	400	2010	0.2	79.4 ± 2.5	22
Sahariah (2012)^1^	Anaerobic	30 ± 2	7.5 ± 0.5	100	200	0.5	90	NA*

One study was found that also measured NO_3_^−^ in both the influent and bioreactor and is presented in the table as a comparison

^1^Sahariah and Chakraborty (2012)

* Data not available

## Electronic supplementary material

Below is the link to the electronic supplementary material.

Supplementary material 1 (XLSX 23 kb)

Supplementary material 2 (XLSX 13 kb)

Supplementary material 3 (DOCX 93 kb)
