# Supplementary material for: Low temperature, autotrophic microbial denitrification using thiosulfate or thiocyanate as electron donor
Source: Biodegradation. 2017 Jun 2;28(4):287–301. doi: 10.1007/s10532-017-9796-7 (PMC5500686; doi:10.1007/s10532-017-9796-7)
Supplement: Supplementary file 3 — Supplementary material 3 (DOCX 93 kb) [file 10532_2017_9796_MOESM3_ESM.docx]

**Low temperature, autotrophic microbial denitrification using thiosulfate or thiocyanate as electron donor**

Elias Broman^1^,* · Abbtesaim Jawad^1^ · Xiaofen Wu^1^ · Stephan Christel^1^ · Gaofeng Ni^1^ · Margarita Lopez-Fernandez^1^ · Jan-Eric Sundkvist^2^ · Mark Dopson^1^

^1^Centre for Ecology and Evolution in Microbial Model Systems (EEMiS), Linnaeus University, Kalmar, Sweden

^2^Boliden Mineral AB, SE-936 8^1^ Boliden, Sweden

**Supplemental Information**

**Supplemental File 1.** Number of read pairs obtained from the sequencing facility, reads remaining after merging, quality trimming and the amount of clustered OTUs.

| **Sequencing facility ID** | **Sample name** | **MiSeq chemistry** | **Read**  **pairs** | **Reads after merging** | **Reads after quality trimming** | **Number**  **of OTUs** |
| --- | --- | --- | --- | --- | --- | --- |
| P933_1095 | Microaerophilic S_2_O_3_^2-^ R3 reactor | PE 2×251 | 129976 | 42820 | 36091 | 43 |
| P933_1096 | Microaerophilic S_2_O_3_^2-^ R3 reactor | PE 2×251 | 109739 | 32660 | 27309 | 46 |
| P1130_2070 | Aerobic Boliden inoculum | PE 2×301 | 54922 | 50352 | 49010 | 168 |
| P1130_2072 | Anaerobic Boliden inoculum | PE 2×301 | 43079 | 40566 | 39520 | 122 |
| P2043_1076 | Aerobic SCN^-^ fed R1 reactor after 231 days (15 ˚C) | PE 2×301 | 138536 | 91179 | 64323 | 95 |
| P2043_1077 | Anaerobic SCN^-^ fed R1 reactor after 231 days (15 ˚C) | PE 2×301 | 107502 | 69493 | 48650 | 104 |
| P2256_1077 | Aerobic SCN^-^ fed R2 reactor after 338 days (10 ˚C) | PE 2×301 | 307995 | 114009 | 72018 | 81 |
| P2256_1078 | Anaerobic SCN^-^ fed R2 reactor after 338 days (10 ˚C) | PE 2×301 | 38868 | 14917 | 9587 | 60 |

**Supplemental File 2.** Pearson correlations of all the chemical parameters measured throughout the experiment (524 days) of the thiocyanate feed bioreactor.

|  | Aerobic (R1) | | | | | | |
| --- | --- | --- | --- | --- | --- | --- | --- |
|  | pH | Redox (mV) | NO_3_^-^ (mg/L) | NO_2_^-^ (mg/L) | COD (mg/L) | SCN^-^ (mg/L) | NH_4_^+^ (mg/L) |
| pH | 1.000 | -0.256 | 0.209 | -0.04 | 0.012 | -0.090 | 0.110 |
| Redox (mV) | -0.256 | 1.000 | -0.137 | -0.207 | 0.060 | 0.107 | -0.274 |
| NO_3_ (mg/L) | 0.209 | -0.137 | 1.000 | 0.170 | -0.103 | -0.270 | 0.596 |
| NO_2_ (mg/L) | -0.04 | -0.207 | 0.170 | 1.000 | -0.334 | -0.416 | 0.519 |
| COD (mg/L) | 0.012 | 0.060 | -0.103 | -0.334 | 1.000 | 0.697 | -0.685 |
| SCN (mg/L) | -0.090 | 0.107 | -0.270 | -0.416 | 0.697 | 1.000 | -0.772 |
| NH_4_ (mg/L) | 0.110 | -0.274 | 0.596 | 0.519 | -0.685 | -0.772 | 1.000 |

|  | Anaerobic (R2) | | | | | | |
| --- | --- | --- | --- | --- | --- | --- | --- |
|  | pH | Redox (mV) | NO_3_^-^ (mg/L) | NO_2_^-^ (mg/L) | COD (mg/L) | SCN^-^ (mg/L) | NH_4_^+^ (mg/L) |
| pH | 1.000 | -0.531 | 0.065 | 0.032 | -0.272 | -0.003 | 0.321 |
| Redox (mV) | -0.531 | 1.000 | 0.193 | -0.300 | 0.358 | 0.228 | -0.521 |
| NO_3_ (mg/L) | 0.065 | 0.193 | 1.000 | -0.203 | 0.542 | 0.329 | -0.280 |
| NO_2_ (mg/L) | 0.032 | -0.300 | -0.203 | 1.000 | -0.267 | -0.530 | 0.774 |
| COD (mg/L) | -0.272 | 0.358 | 0.542 | -0.267 | 1.000 | 0.468 | -0.686 |
| SCN (mg/L) | -0.003 | 0.228 | 0.329 | -0.530 | 0.468 | 1.000 | -0.617 |
| NH_4_ (mg/L) | 0.321 | -0.521 | -0.280 | 0.774 | -0.686 | -0.617 | 1.000 |

**Pearson correlations** (*n* = 102)

p < 0.01

p < 0.05

**Supplemental File 3.** Optical density and COD measurements from the aerobic R1 (light grey circles) and anaerobic R2 (dark grey circles) SCN^-^ fed bioreactor adapted from 21°C room temperature to 8°C over a period of 338 days. The pH was lowered from 8.0-8.5 to 5.5 on day 448 and further lowered to pH 3.8 on day 510.


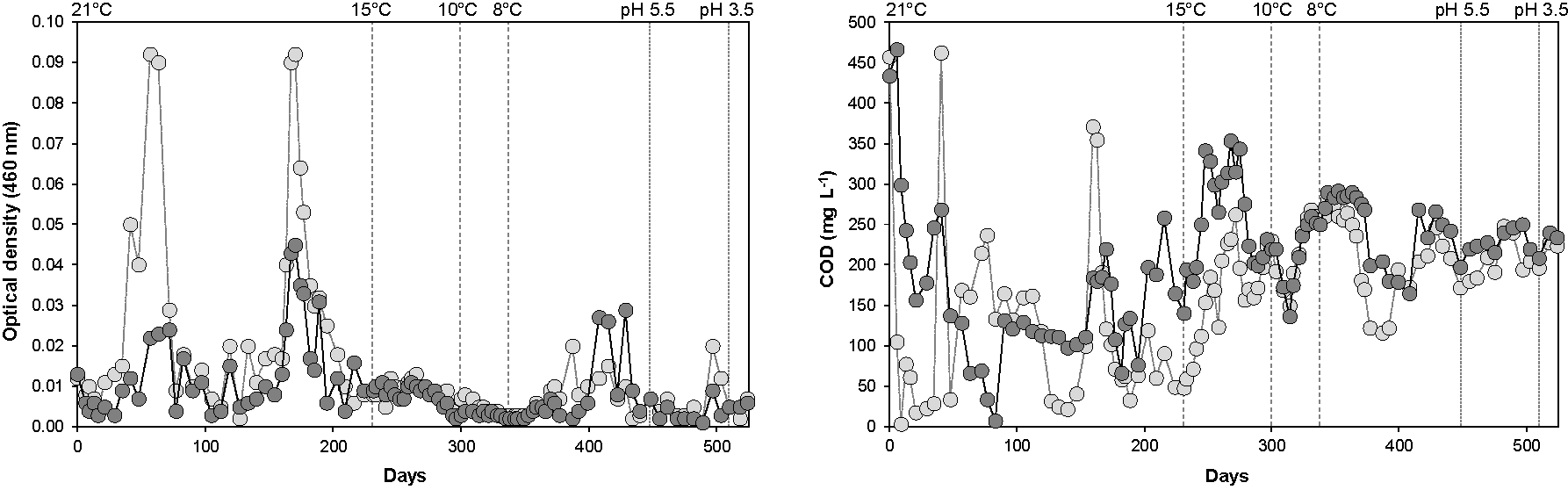


**Supplemental File 4.** Results from the annotation against the SILVA database for the thiocyanate fed bioreactors. The top header shows the sequencing facility ID, while the row below shows the sample name. Relative OTU abundance is shown.

**Supplemental File 5.** Protein and redox potential measurements from the microaerophilic S_2_O_3_^2-^ fed R3 bioreactor at 8°C.


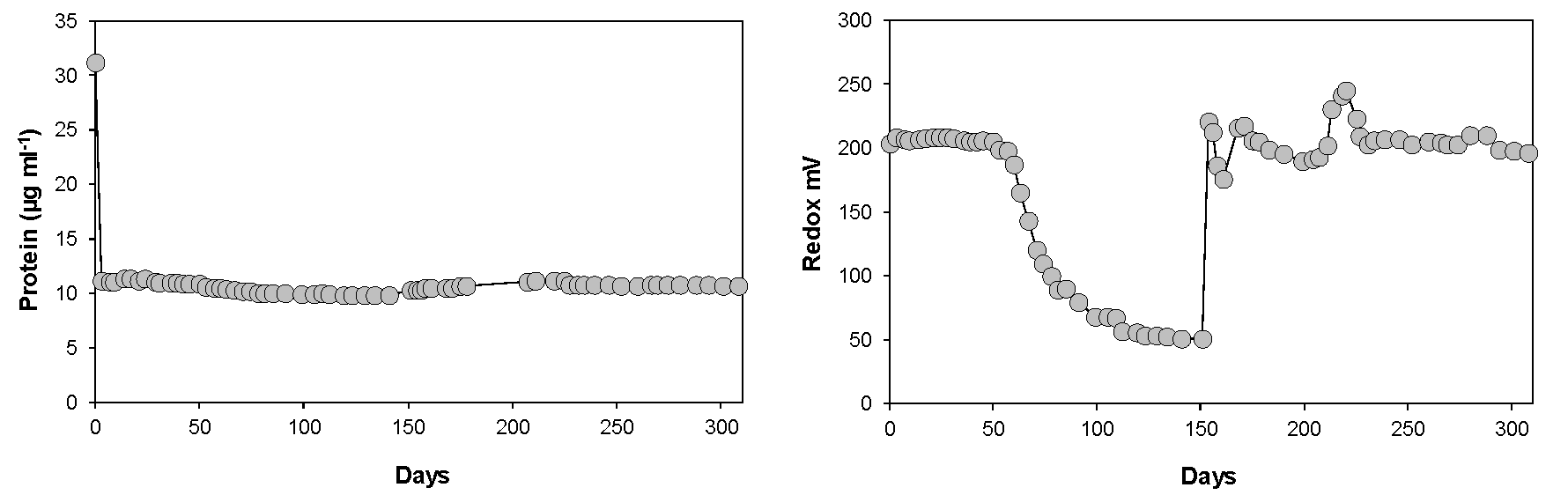


**Supplemental File 6.** Results from the annotation against the SILVA database for the thiosulfate fed R3 bioreactor (two technical replicates). The top header shows the sequencing facility ID, while the row below shows the sample name. Relative OTU abundance is shown.

**Supplemental File 7.** Potential reactions in the thiocyanate bioreactors.

Aerobic R1 bioreactor

2 H_2_O + SCN^-^ + 2 O_2_ → NH_4_^+^ + CO_2_ + SO_4_^2-^ uu (1)

H_2_O + SCN^-^ + 2 O_2_ → CNO^-^ + SO_4_^2-^ + 2 H^+^ (2)

CNO^-^ + H_2_O + 2 H^+^ → CO_2_ + NH_4_^+^ (3)

4 NO_3_^-^ + SCN^-^ + 2 H_2_O → 4 NO_2_^-^ + NH_4_^+^ + CO_2_ + SO_4_^2-^ (4)

1.6 NO_3_^-^ + SCN^-^ + 1.6 H^+^ + 1.2 H_2_O → NH_4_^+^ + 0.8 N_2_ + CO_2_ + SO_4_^2-^ (5)

NH_4_^+^ + 1.5 O_2_ +H_2_O → NO_2_^-^ + 2 H^+^ + H_2_O (6)

NO_2_^-^ + O_2_ → NO_3_^-^  (7)

Anaerobic R2 bioreactor

2 H_2_O + 10 SCN^-^ + 16 NO_3_^-^ → 10 CNO^-^ + 8 N_2_ + 10 SO_4_^2-^ + 4 H^+^ (8)

12 H_2_O + 10 SCN^-^ + 16 NO_3_^-^ + 16 H^+^ → 10 CNO^-^ + 8 N_2_ + 10 CO_2_ + 10 NH_4_^+^ + 10 SO_4_^2-^ (9)
